# Fast Identification of Bacteria for Quality Control of Drinking Water through a Static Headspace Sampler Coupled to a Sensory Perception System

**DOI:** 10.3390/bios9010023

**Published:** 2019-02-08

**Authors:** Jeniffer Carrillo, Cristhian Durán

**Affiliations:** GISM Group, Faculty of Engineering and Architecture, University of Pamplona, Pamplona–North of Santander, Colombia; cmduran@unipamplona.edu.co

**Keywords:** electronic nose, *E. coli*, sensors, quality control, drinking water, pattern recognition

## Abstract

The aim of this study was to develop and implement a methodology composed by a Static Head-Space-Sampler (SHS) coupled to a Sensory Perception System (SPS) for the extraction of Volatile Organic Compounds (VOC’s) emitted by bacterial species in the water. The SPS was performed by means of a chamber of 16 Metal-Oxide-Semiconductor (MOS) gas sensors and a software with pattern recognition methods for the detection and identification of bacteria. At first, the tests were conducted from the sterile and polluted water with the *Escherichia coli* bacteria and modifying the incubation temperatures (50 °C, 70 °C and 90 °C), with the objective to obtain an optimal temperature for the distinguishing of species. Furthermore, the capacity of the methodology to distinguish the important compounds was assessed, in this case, *E. coli* and other bacteria like *Pseudomonas aeruginosa* and *Klebsiella oxytoca*, which formed similar analytes. The validation of the proposed methodology was done by acquiring water samples from different unitary operations of an aqueduct of the municipality of Toledo (North of Santander, Colombia), which were analyzed by the membrane filter technique in the laboratories of the University of Pamplona, along with the SHS-SPS system. The results showed that it was possible to distinguish polluted water samples in a fast way through the sensory measurement equipment using pattern recognition techniques such as Principal Component Analysis (PCA), Discriminant Function Analysis (DFA) and a probabilistic neural network (PNN), where a 95% of differentiation was obtained through PCA and 100% of the classification with DFA. The PNN network achieved the 86.6% of success rate with the cross-validation technique “leave one out”.

## 1. Introduction

The on-line monitoring of water for human consumption resulting from drinking-water treatment plants or aqueducts is an important tool to evaluate water plant operating efficiency and implement the operational parameters to produce a high-quality water which achieves regulation exigencies.

Nowadays, drinking-water conditioning is a process that involves the realization of certain physicochemical and microbiological parameters that are regulated with the current guidelines of water quality. At the international level, the World Health Organization (WHO) determines that suitable water for human consumption has to be free of any microorganism which may affect consumer’s health. This parameter is used as a quality guide for the rules or standards creation. Also, it aids to evaluate the contamination risk to monitor the treatment processes and apply corrective plans to achieve the desired quality [1]. In Colombia, the microbiological quality includes bacteria control such as total and fecal coliforms in which the permissible values are contained in the 2115/2007 resolution, and 25 articles indicating that the specified value for these microorganisms is zero for each 100 ml when it comes to water for human consumption. Additionally, in this normativity are stipulated in the different conventional methods (fermentation of multiple tubes, membrane filtration, enzyme substrate, defined substrate, among others) used for bacteria detection. However, it is important to highlight that they are complex methods and represent high costs and consumption time for the analysis, because they are a challenge to use for establishing the microbial water quality for human consumption in a specific time.

Currently, one way of verifying the microbiological quality of the water is by constantly monitoring a bacterial indicator as important as *Escherichia coli* [2], however, it is a big limitation at the moment, for analyzing this bacteria in the majority of treatment drinking-water plants, taking into account that they do not have good certified laboratories, specialized technologies and nor trained personal, to be able to perform the mentioned microbiological tests limiting the frequency of analysis (once to twice a month). Besides, the samples have to be sent to certified laboratories generating costs, and faults derived from the sampling can modify the analysis and delays results. That is the reason why the limitations of the conventional methods have caused investigations to focus on the development of fast and precise methods for bacteria detection.

For a continuous monitoring with fast response times and low cost, new measurements techniques have been developed through Electronic Noses (E-noses), given that they are an alternative instrumental measurement, replacing the conventional methods used for the detection and bacteria identification in different types of samples. Some of them are cited below: [3,4,5,6,7,8,9]. According to the investigations carried out during the last years about the electronic noses, it is important to mention that this equipment can be used for specific analysis, identification and recognition of complex smells and volatile organic compounds since it is composed of a group of chemical gas sensors with partial sensibilities [10]. Nevertheless, as a general rule, it cannot afford to ignore the re-conditioning stage of the sample for the analysis, because the sample preparation is an important factor in the analytical process and it has to be as simple as possible (fast response without using additional complex instruments). On the other hand, in the sampling it is necessary to obtain a sample in the steam phase (represents the characteristic of the compound under research) which must be in contact with the sensors array with good sensitivity and selectivity. Also, it must be representative in relation to the specific bacterial species compounds that form the sample as well as to improve the classification and strength quality of the measurement. A way to increase the sensitivity and selectivity is to integrate sample concentration methods, for this, there exists techniques such as the Solid Phase Micro-Extraction (SPME) [11,12], and dynamic and static Headspace (HS) Techniques. The static HS analysis is the most popular and simple sample preparation technique to be used with gas sensors, because it does not require special instrumentation nor specialized staff for any additional procedures, in addition, it is low-cost. The sensory perception system functioning is conducted by the development and implementation of algorithms used as patterns recognition methods, such as: Principal Component Analysis (PCA), Discriminant Function Analysis (DFA) and the Probabilistic Neural Networks (PNN), which allow researchers to discriminate and classify the compounds generated by the different samples.

## 2. Materials and Methods

**Figure 1:**
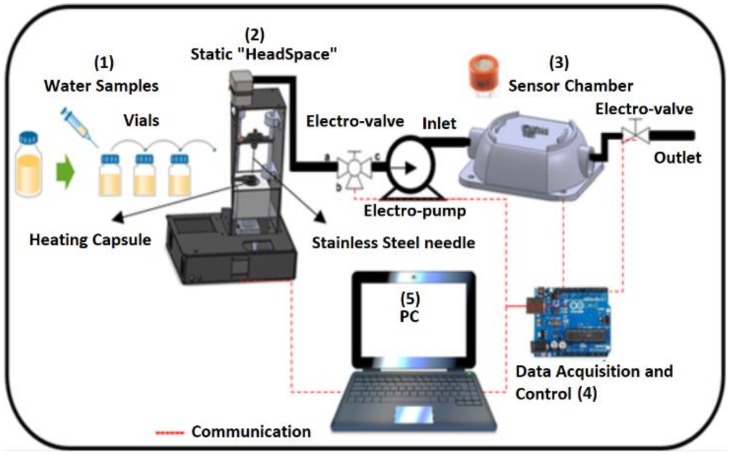
General scheme of the methodology.

Figure 1 describes each one of the phases that were carried out for the developed methodology: (1) Preparation and samples conditioning (vials), (2) static “HeadSpace”, (3) sensor chamber, (4) a data acquisition card to store the information obtained from the sensors chamber and (5) PC.

### 2.1. Static HeadSpace Sampler (SHS) 

The SHS system was based on vial temperature control through a Proportional- Integral- Derivative (PID) controller for the proportional activation of a heating capsule and the operation of a stepper motor for control of the injector in an automatic way. The process begins when locating the liquid sample which comprises the organic analytes in a 20 ml hermetically sealed vial through a septum that was located in the heating cell. Through a screen type Liquid Crystal Display (LCD) the time and the incubation sample temperature (ranging from 0 °C to 110 °C) were defined with the aim to generate volatile components (HeadSpace) and later to perform the extraction of those compounds.

**Figure 2:**
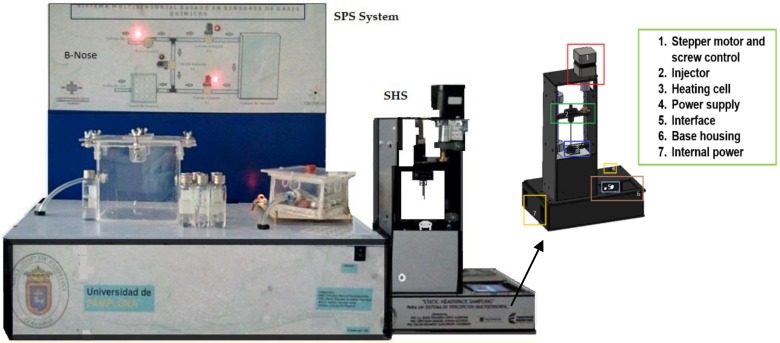
SHS system coupled to SPS (B-NOSE).

A worm screw was driven automatically by means of the stepper motor that shifts a stainless-steel needle from the top down in order to remove the volatile compounds from the vial. In this step it was necessary to activate an electric motor and a three-way electro-valve by a determined time that allows pulling all the volatile components generated before towards the entry of the measuring chamber. Figure 2 illustrates the materials used to perform the SHS system analysis. 

### 2.2. Sensory Perception System

In this research a SPS called B-NOSE manufactured at Universidad of Pamplona (Colombia) with 16 metal oxide gas sensors made by Figaro sensor company, was implemented, which was developed at University of Pamplona (2011). It is noted that all sensors were selected in a non-specific way to obtain a high spectrum, in order to identify or classify the water samples. Table 1 illustrates the characteristics of the sensors. 

It is important to mention that five duplicate-sensors (i.e., TGS 826, TGS 831, TGS 821, TGS 842 and TGS 880) were placed inside of the sensor chamber since they showed a very good sensitivity and selectivity. Although these duplicate-sensors belonged to the same model, their responses were different, therefore very useful and reliable information was obtained in the data set in order to achieve the expected results. Normally, these types of gas sensors are manufactured by means of a resistive sensing material, for this reason the resistance value changes and its responses will never be equal to each other. Furthermore, the calibration process and the sensor chamber purge were realized in the same way through environmental air.

### 2.3. Sample Conditioning

In this study three different types of samples were evaluated: (1) The negative control: It consisted of sterile water (autoclaved for about 15 minutes, at a temperature of 121.1 °C), (2) the positive sample: It was prepared using sterile water inoculated with *Escherichia coli* (strain ATCC 25922) grown on nutritive agar at 37 °C for 18–24 h, and finally (3) the specificity controls, which were prepared with sterile water samples contaminated in a controlled way with *Pseudomonas aeruginosa* (ATCC 27853) and *Klebsiella oxytoca* (ATCC 49131) bacteria. In this research it was necessary to take into account that in the preparation of three bacterial suspensions, isolated colonies of each bacteria were used with the same culture medium for their growth.

For the preparation of each one of the samples (positive and specificity control) a bacterial suspension was used at a concentration of 3 × 10^8^ Colony Forming Unit (CFU)/cm^3^, using as a reference the standard No. 1.0 of the “McFarland Scale”. Subsequently, this bacterial suspension was diluted 30 times in sterile water to obtain 300 ml of both the positive control and the specificity controls, with a final concentration of 1 × 10^7^ CFU/cm^3^. After the dilution, the samples were incubated for around 12 h at 37 °C, in order to improve the enrichment of the compounds generated. 

### 2.4. Measurements

As a first stage, 20 samples of sterile water and *Escherichia coli* were analyzed modifying the incubation temperature on the vials (i.e., At 50 °C an amount of 20 samples were taken, in the same way an amount of 20 samples were taken at 70 °C and 90 °C); it was done in order to verify the optimum temperature at which the SHS-SPS discriminates between the negative control and the sample with the analyte (*E. coli*). Once the first stage was completed and according to the results obtained, the optimal temperature (i.e., 50 °C) was chosen to evaluate the ability of SHS-SPS to distinguish between *E. coli* and the other analytes that are similar and may be confounding factors.

Therefore, *Pseudomonas aeruginosa* and *Klebsiella oxytoca* bacteria belonging to the same family as *E. coli* were selected as specificity controls. The results obtained were compared with sterile water samples (negative control). For the validation of the implemented methodology, water samples were taken from the drinking-water treatment plant of the Municipality of Toledo (North of Santander-Colombia) at 4 different points: (a) Entrance of the plant (parshall flume), (b) after sedimentation, (c) output of the plant and (d) tap water. The samples collected were divided into two groups: The first group refers to the samples that were analyzed in the microbiology laboratories at the University of Pamplona (UP) by means of the membrane filtration method and the second group corresponds to the samples analyzed by the methodology implemented.

### 2.5. Data Processing

The pre-processing and data processing were carried out through the use of parameter extraction, normalization and pattern recognition methods. Initially, the static parameter (ΔG) was obtained to get the relevant information from the data set (ΔG = G_max_ − G_min_), where G_max_ is the maximum conductance of the sensor response and the G_min_ parameter is the basis point of the minimum conductance value of the sensor. Afterwards, they were normalized applying the following normalization methods: auto-scaling, centered data and matrix normalization. Table 2 shows each normalization method and formula, where $X_{m}$ denotes the response of sensors in the database and $Y_{m}$, the normalized response. 

As previously mentioned, the data processing was conducted with PCA analysis (discrimination method), DFA (classification method) and an alternative classification method was applied with the probabilistic neural network (PNN), which was implemented with the cross-validation technique called “leave one out”.

## 3. Results and Discussion

**Figure 3:**
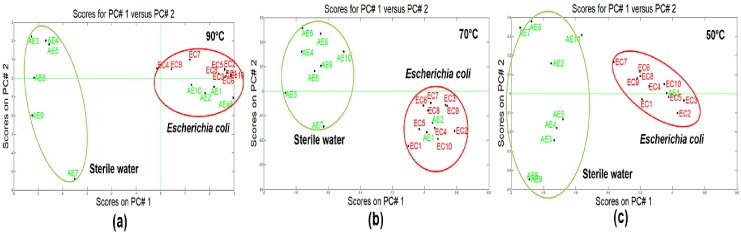
Plotted PCA of sterile water and *Escherichia coli* a: (**a**) 90 °C, (**b**) 70 °C and (**c**) 50 °C.

Figure 3a–c illustrate the results of applying the PCA analysis where can it be observed the differentiation between two categories: *E-coli* (red tag) and sterile water (green tag), regardless of the incubation temperature. However, some overlapped sterile water samples were obtained whereas analyzing the effect in the sample temperature, it was concluded that this has an important role in the repeatability of the measurement results and differentiation of the studied analytes. 

According to the research made by Roussel et al [13], they explained that in high temperatures the VOC’s enrichment can be enhanced by increasing the concentration to be extracted and analyzed, improving the sensors sensitivity and the results. Similarly, sometimes a great inconvenience appeared which depending on the sample origin in high temperatures, it can be decomposed leading to unfavorable results. In this research, based on the obtained results it was evidenced that the best analytic differentiation was obtained at low temperatures, in this case the optimum classification temperature was fixed at 50 °C, getting a variance of 98.7% regarding the differentiation obtained by means of percentages of other temperature: 70 °C (97.8%) and 90 °C (96.1%).

**Figure 4:**
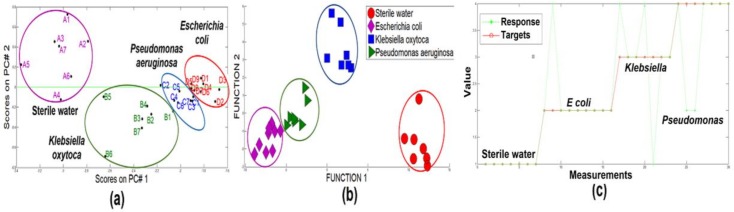
Plot Analysis: (**a**) PCA (differentiation); (**b**) DFA (Classification) and (**c**) PNN (classification (leave-one-out).

In Figure 4a, a good cluster differentiation can be observed between different categories by using PCA (97.9% of variance). It was considered the case in which all the samples of the three bacterial species and the sterile water were combined in one data group (7 sterile water samples, 7 *Pseudomonas aeruginosa*, 7 *Klebsiella oxytoca* and 9 *Escherichia coli*). The most interesting characteristic is that a separation among these three categories and the sterile water could be determined when the DFA was applied (Figure 4b), obtaining a 100% of success rate for classification. PCA analysis also confirms that SHS-SPS responses for the *E. coli* samples are not very different to the *Pseudomonas* and *Klebsiella* samples; besides, there are some researches that have identified a certain grade of specificity in terms of volatile organic compounds characteristic of each type of bacteria that is common in the interest species, due to shared metabolic pathways. The “green lines” belong to the neural network response and the solid line “red color” belongs to the targets. By using the data training and cross validation technique “leave-one-out”, a success rate of 86.6% classification was obtained with only four unclassified measurements (see Figure 4c). 

**Figure 5:**
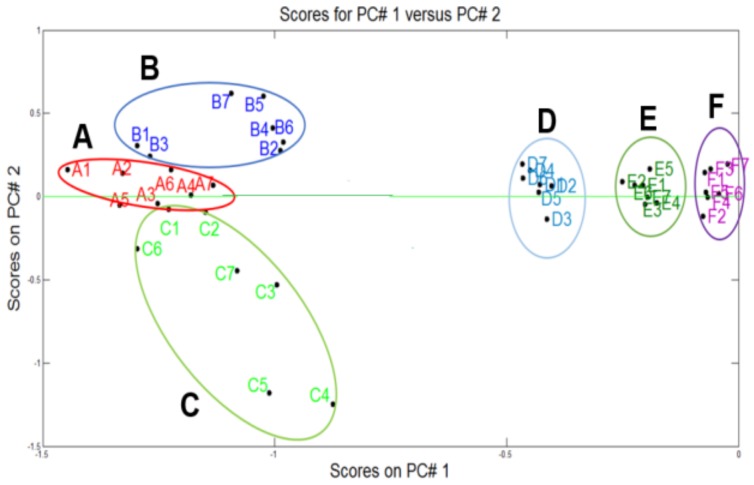
(**A**) Sterile water, (**B**) Output water of the aqueduct, (**C**) Tap water, (**D**) Water from sedimentation tank, (**E**) Input water of the aqueduct y (**F**) *Escherichia coli*.

Figure 5 depicts the differentiation among the clusters. On the left part of the plot, the non-contaminant samples (sterile water, output water and tap water) were discriminated and on the right part, the samples from the sedimentation tank and the input water were grouped very close to the samples inoculated with the *E. coli* bacteria. 

According to the PCA model of Figure 3c where the sterile water (A) and *E.coli* (F) were discriminated at a concentration of 1 × 10^7^ CFU/cm^3^, a third test was carried out, which consisted of collecting samples from the Drinking Water Treatment Plant (DWTP), (B, C, D and E) with the aim to project them into the new PCA model to detect the presence of *E. coli* in the water samples.

To validate the results obtained in the previous figure, the identification and classification of the bacterial pollution of the samples taken from the plant were analyzed using the membrane filter technique. According to the microbiological requirements demanded by the 2115 resolution and guidelines, they are considered as *E. coli* polluted samples those who exceed the indicator value taken as the acceptability limit (0 CFU/cm^3^ water). 

Table 3 shows the microbiological analysis of the input water and the sedimentation tank samples of the plant in Colony-Forming Units (CFU), where the results of the polluted samples with *E. coli* gave concentrations of 125 CFU/cm^3^ and 26 CFU/cm^3^ respectively. Regarding the output water of the plant and tap water, they did not present bacteria incidence (0 CFU/cm^3^), indicating the efficiency in the treatment process. 

It should be noted that this evaluation was only done to determine the efficiency of the SHS-SPS with regard to the microbiological analysis using membrane filtration method to detect the water contaminated with *E. coli*. In this case the concentration was not calculated nor compared. 

For the identification of volatile compounds present in *Escherichia coli* bacteria (strain ATCC 25922) a database of microbial volatiles called *“mVOC”*, was used on this study [14]. This database lists more than one thousand microbial volatile compounds and contains information about bacteria. Table 4 illustrates the information of microbial volatile compounds emitted by *E. coli*.

**Figure 6:**
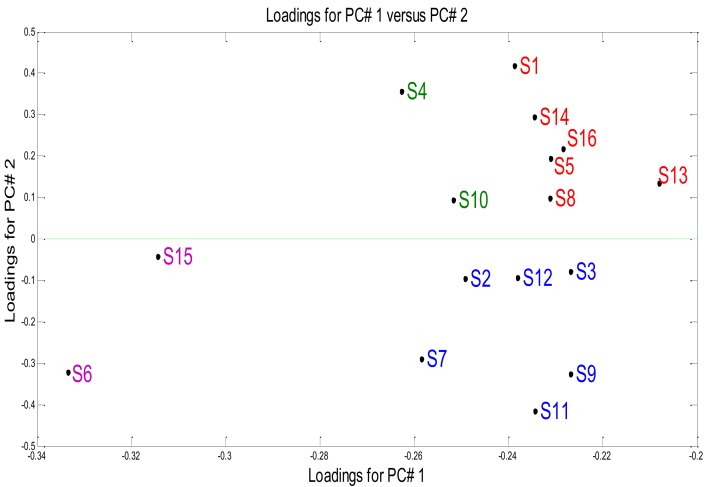
Loadings of bacteria: (Purple) Sterile water, (Red) *E. coli*, (Blue) *Pseudomonas aeruginosa* and (Green) *Klebsiella oxytoca*.

It is important to highlight that all sensors responded to the contaminated water and non-contaminated samples. The contribution of each sensor to bacterial discrimination from a set of measurements, depended on the gas concentration (Parts per million (ppm)). Figure 6 illustrates the contribution of the sensors to the samples’ discrimination and mentioned bacteria above, applying the PCA loadings, where the most discriminating sensors were sensitive. The loadings are the contribution of each original variable when calculating the principal component. 

Regarding the samples detection, the loadings of the original variables TGS831 (S15) and TGS880 (S6) sensors were sensitive to the halocarbons that its related to the sterile water. TGS826 (S1), TGS842 (S5), TGS813(S8), TGS832(S13), TGS842(S14) and TGS830(S16) sensors responded the highest to alkenes, ketones that were related to *E. coli*. On the other hand, TGS821 (S3), TGS800 (S9), TGS822 (S11), TGS831 (S2), TGS825(S7) and TGS821 (S12) sensors were more sensitive to toxic gas detection as well as alcohol that were referred to as *Pseudomonas aeruginosa* and with regard to TGS826(S4) and TGS880(10) sensors, detection of *Klebsiella oxytoca* was referred to as odor detection.

## 4. Conclusions

According to the measurements between the sterile water and *E. coli* at different temperatures, it the good performance of the methodology and repeatability of the measurements was demonstrated, obtaining more than 90% of the differentiation of these species. The results showed a good classification of 100% with DFA and through the PNN network using the cross-validation technique, where the success rate percentage was 86.6%, which indicates that the proposed method was able to extract and detect the characteristic compounds of each sample group, allowing the differentiation between each bacterial species and the sterile water. In this study, it was also possible to discriminate and classify among different types of contaminants (*E. coli* and other bacteria such as: *Pseudomonas aeruginosa* and *Klebsiella oxytoca*) using a e-nose system composed of a gas sensor array. 

On the other hand, it is very important take into account the concept of an e-nose which is based in a partially overlapping sensitivity obtained with a sensor array. The sensor sensitivity depends of the type of gas, the time and the VOC concentration. The e-nose is a very useful technology since in comparison with gas chromatography–mass spectrometry (GC-MS), this artificial system can detect a smell-print or an overall compound using a simple sensor array; it is an “all or nothing” process. Furthermore, with this methodology is not necessary to specify the type of compounds in water; only a good discrimination and selectivity of all compounds give a reliable response. For this reason, a short-term aim might be that the proposed method coincides with the sensitivity and the specificity of the conventional methods (GC-MS) used for the identification and classification of bacteria. Under this motivation, in future work the results obtained in this study will be compared with the membrane filter technique and the GC-MS analysis. Nevertheless, it is important to highlight that in this study was validated the specificity of the system by evaluating the capacity of the methodology SHP-SPS to differentiate the compounds or analytes of interest, in this case, the *E. coli* bacteria with others with similar conditions.

The results achieved with this tool are promising because thanks to its portability, sensitivity, rapidity and low cost, it is possible to take the respective corrective and preventive actions in an aqueduct and thus produce high-quality water for human consumption, fulfilling the demands of normativity.

## Figures and Tables

**Table 1:** Gas sensors.

#	Sensor	Amount	Application	Type of Sensible Heat
1	TGS 826	2	Odor detection	Ammoniate and amine
2	TGS 831	2	Halocarbons gases detection (refrigerant gases)	Chlorofluorocarbons (CFC)
3	TGS 821	2	Combustible gas detection	Hydrogen
4	TGS 842	2	Combustible gas detection	Methane and natural gas
5	TGS 880	2	Cooking control	Smoke of the food (Alcohol, odor)
6	TGS 825	1	Toxic gas detection	Hydrogen sulfide
7	TGS 813	1	Combustible gas detection	Hydrocarbons in general
8	TGS 800	1	Air quality monitoring	Air pollutants in general
9	TGS 822	1	Solvent vapors detection	Alcohol and organic solvents
10	TGS 832	1	Halocarbons gases detection (refrigerant gases)	R-134 1,1,1,2-Tetrafluoroethane
11	TGS 830	1	Halocarbons gases detection (refrigerant gases)	R-22 Monoclorodifluoromethane

**Table 2:** Normalization methods.

Normalization Method	Formula
Auto-scaling	$Y_{m}=\frac{X_{m}-mean\left[X_{m}\right]}{std\left[X_{m}\right]}$
Mean-centering	$Y_{m}=X_{m}-mean\left[X_{m}\right]$
Matrix normalization	$Y_{m}=\frac{X_{m}}{\max\left[X_{m}\right]}$

**Table 3:** Microbiological analysis using the membrane filtration method.

Analysis	Input Water	Sediment-Water	Output Water	Tap Water	Normativity
*E. coli*	125 CFU/cm^3^	26 CFU/cm^3^	0 CFU/cm^3^	0 CFU/cm^3^	0 CFU/cm^3^

**Table 4:** Microbial volatile compounds emitted by *E. coli*.

Microbial Volatiles Name	Formula	Chemical Classification
Decan-1-ol	C_10_H_22_O	Alcohol
Dodecan-1-ol	CH_3_(CH_2_)_10_CH_2_OH	Alcohol
Nonan-2-one	C_9_H_18_O	Ketone
Octan-1-ol	C_8_H_18_O	Alcohol
Tridecan-2-one	C_13_H_26_O	Ketone
Undec-1-ene	C_11_H_22_	Alkenes
Undecan-2-one	C_11_H_22_O	Ketone
